# The value of CT, MRI, and PET-CT in detecting retropharyngeal lymph node metastasis of head and neck squamous cell carcinoma

**DOI:** 10.1186/s12880-020-00487-y

**Published:** 2020-07-29

**Authors:** Jin Hwan Kim, Kyu Young Choi, Sang-Hyo Lee, Dong Jin Lee, Bum Jung Park, Dae Young Yoon, Young-Soo Rho

**Affiliations:** 1 Department of Otorhinolaryngology-Head and Neck Surgery, Kangnam Sacred Heart Hospital, Hallym University College of Medicine, Daerim-1dong, Yeongdeungpo-gu, Seoul, 150-950 South Korea; 2 Department of Radiology, Hallym University College of Medicine, Seoul, South Korea

**Keywords:** Tomography, X-ray computed, Magnetic resonance imaging, Positron-emission tomography, Head and neck neoplasms, Carcinoma, squamous cell, Lymph nodes, Lymphadenopathy

## Abstract

**Background:**

The diagnostic accuracies of the imaging studies should be clearly acknowledged in managing head and neck cancer patients; however, the accuracies of preoperative imaging studies in detecting retropharyngeal lymph node (RPLN) metastasis are still not clarified. This study was to evaluate diagnostic accuracies of computed tomography (CT), magnetic resonance imaging (MRI), and positron emission tomography-computed tomography (PET-CT) in detecting RPLN metastasis of head and neck squamous cell carcinomas.

**Methods:**

For 123 patients who had performed RPLN dissection during the surgery of their squamous cell carcinoma of the head and neck, preoperative CT, MRI, and/or PET-CT were reviewed for RPLN metastasis in a blinded fashion by one experienced radiologist. Sensitivity, specificity, positive predictive value, negative predictive value, and overall accuracy of each imaging modality were assessed, by comparing with the histopathologic findings of the resected RPLNs that served as the standard of reference.

**Results:**

RPLNs were pathologically positive for metastasis in 43 of the 123 patients (35%). Sensitivity, specificity, positive predictive value, negative predictive value, and overall accuracy in detecting metastasis to RPLN were 65, 94, 85, 83, and 84% for CT; 74, 94, 87, 87 and 87% for MRI; 83, 93, 89, 89 and 89% for PET-CT, respectively. When all the three imaging modalities were considered together (*n* = 74), they offered sensitivity of 90%, specificity of 91%, positive predictive value of 87%, negative predictive value of 93%, and accuracy of 91%.

**Conclusions:**

The preoperative imaging studies offered relatively high specificity rates, but rather low sensitivity rates. The three imaging modalities altogether increased diagnostic accuracies, which highlights the potential of the three studies when used altogether can minimize missed diagnoses of RPLN metastasis.

## Background

Tumor metastasis to the retropharyngeal lymph nodes (RPLNs) is as a strong predictor of poor prognosis in head and neck cancer patients [1–4]. The most common primary tumor giving rise to RPLN metastasis is nasopharyngeal carcinoma, for which the RPLNs are routinely included in the treatment field [5]. However, RPLNs are involved in up to 20–50% of oropharyngeal, hypopharyngeal, and cervical esophageal carcinomas [1, 6–8].

The retropharyngeal space cannot be easily accessed in physical examinations of the head and neck, so understanding of the RPLN status in patients with head and neck cancer relies heavily on radiologic studies. Contrast-enhanced computed tomography (CT) is commonly used to identify lymph node metastasis; however, given the low sensitivity of CT, magnetic resonance imaging (MRI) is often used together with CT to stage cervical lymph nodes [9]. While MRI and CT are the most commonly used approaches for assessing RPLN involvement, positron emission tomography–computed tomography (PET-CT) in combination with CT or MRI increases sensitivity and specificity of these techniques [5, 9]. Patients with RPLN metastasis are known to have poor prognosis [1–4] so the accuracy of preoperative imaging studies should be fully taken into account; however, limited data are available and the reported accuracies of these studies in determining the presence or absence of metastatic RPLNs are inconsistent [1, 2, 9, 10]. The purpose of this study was to evaluate the diagnostic accuracies of CT, MRI, and PET-CT in determining RPLN metastasis by comparing their results with pathology results in patients with squamous cell carcinomas of the head and neck who underwent RPLN dissection.

## Methods

### Patients

Patients who had head and neck cancer surgery that included RPLN dissection, and were finally diagnosed with squamous cell carcinoma of the head and neck pathologically were consecutively selected in a university hospital cancer center (Ilsong Memorial Institute of Head and Neck cancer, Hallym University Medical Center, Seoul, Korea). For the revision cases, only the patients who had their prior treatment (surgery, chemotherapy or radiotherapy) at least 1 year before the RPLN dissection were included. From December 2006 to December 2014, 134 patients who met the above criteria were found. All the patients had taken both CT and MRI as a staging work-up preoperatively, but the retropharyngeal area was not adequately investigated by CT in 11 cases due to dental artifacts. After excluding these 11 cases, a total of 123 patients were finally included in the study. Among them, 74 patients had taken preoperative PET-CT. Retrospective review of medical records was carried out for demographic data, primary site and histopathology of the cancer, tumor-node-metastasis (TNM) stage (according to the criteria of the 2009 American Joint Committee on Cancer, 7th edition) [11], and the pathology reports of the dissected RPLNs. Preoperative imaging studies (CT, MRI, and PET-CT) were reviewed in a blinded fashion by one experienced radiologist (DYY who have been specializing in head and neck imaging for 12 years), who then determined whether the results were positive or negative for cancer metastasis to the RPLNs. The study was approved by the Institutional Review Board of the Clinical Research Institute at Hallym University Hospital, and the individual consents were waived due to the retrospective study design.

### Imaging analysis

All the patients had taken a spiral CT scan (LightSpeed, General Electric Medical Systems, Milwaukee, WI, USA). Axial CT scans with contrast, taken at 3 mm intervals from the base of the skull to the carina were reviewed. Contrast-enhanced CT was obtained 90–100 s after intravenous administration of 80–100 mL of iodinated contrast material at a rate of 1 mL/s. MRI was performed using a 1.5-T MR imaging system (Philips Gyroscan, Eindhoven, Netherlands). The imaging protocol included T2-weighted fast spin-echo images with fat suppression (repetition time of 2000–2800 ms, echo time of 80–120 ms, 3 mm slice thickness with no inter-slice gap), T1-weighted spin-echo images (repetition time of 425–500 ms, echo time of 12–15 ms, 3 mm slice thickness with no inter-slice gap), and contrast-enhanced T1-weighted spin-echo images following a bolus injection of 0.1 mmol/kg gadolinium dimeglumine (Schering AG, Germany) with the use of a 512 matrix. The combined PET-CT scanner used was a Discovery LS (General Electric Medical Systems) which consisted of a PET scanner with bismuth germanate crystal detectors and a LightSpeed multi-slice helical CT scanner housed together. After a 6-h fasting period, patients were injected intravenously with 12–15 mCi of ^18^fluorine-fluorodeoxyglucose (FDG), and images were acquired 60-min later. A scout view was first taken, followed by a spiral CT scan with 0.8-s rotation time and 4-mm section thickness. This scan was followed by acquisition of PET emission images; the CT data were used for attenuation correction of PET emission images.

**Fig. 1:**
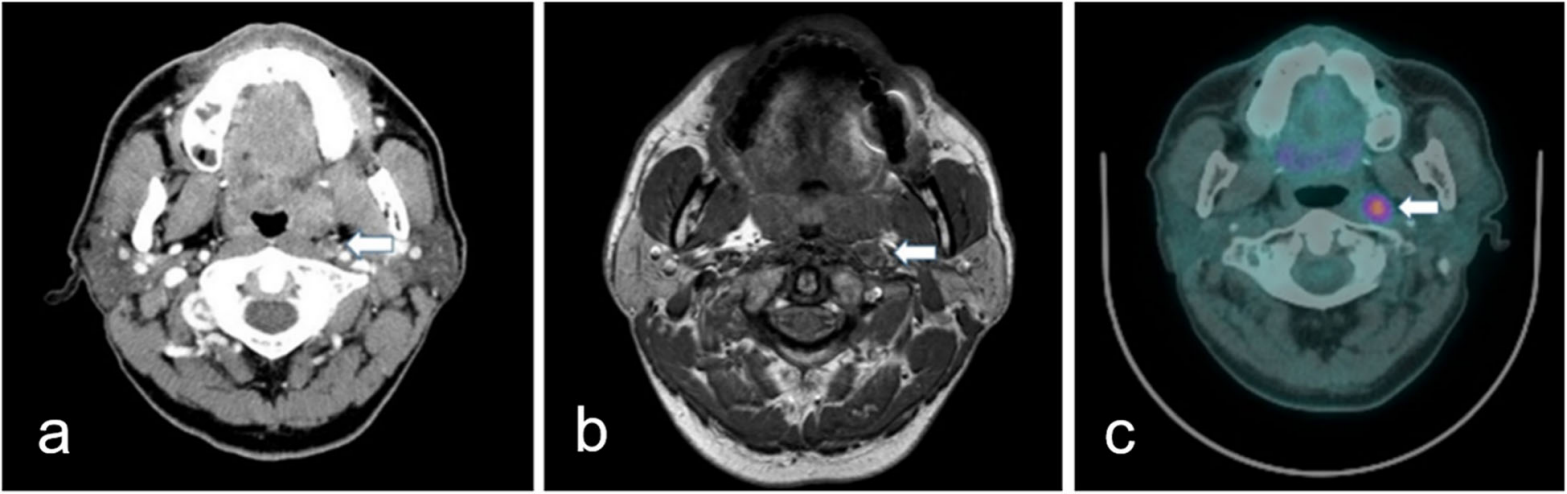
Representative true positive imaging findings of retropharyngeal lymph node. **a** Contrast-enhanced computed tomography shows a left metastatic retropharyngeal lymph node (*arrow*). **b** T1-weighted gadolinium-enhanced magnetic resonance imaging shows a left metastatic retropharyngeal lymph node (*arrow*). **c** Positron emission tomography-computed tomography shows an abnormal ^18^fluorine-fluorodeoxyglucose uptake with a standardized uptake value of 3.1, on a left retropharyngeal lymph node (*arrow*)

**Fig. 2:**
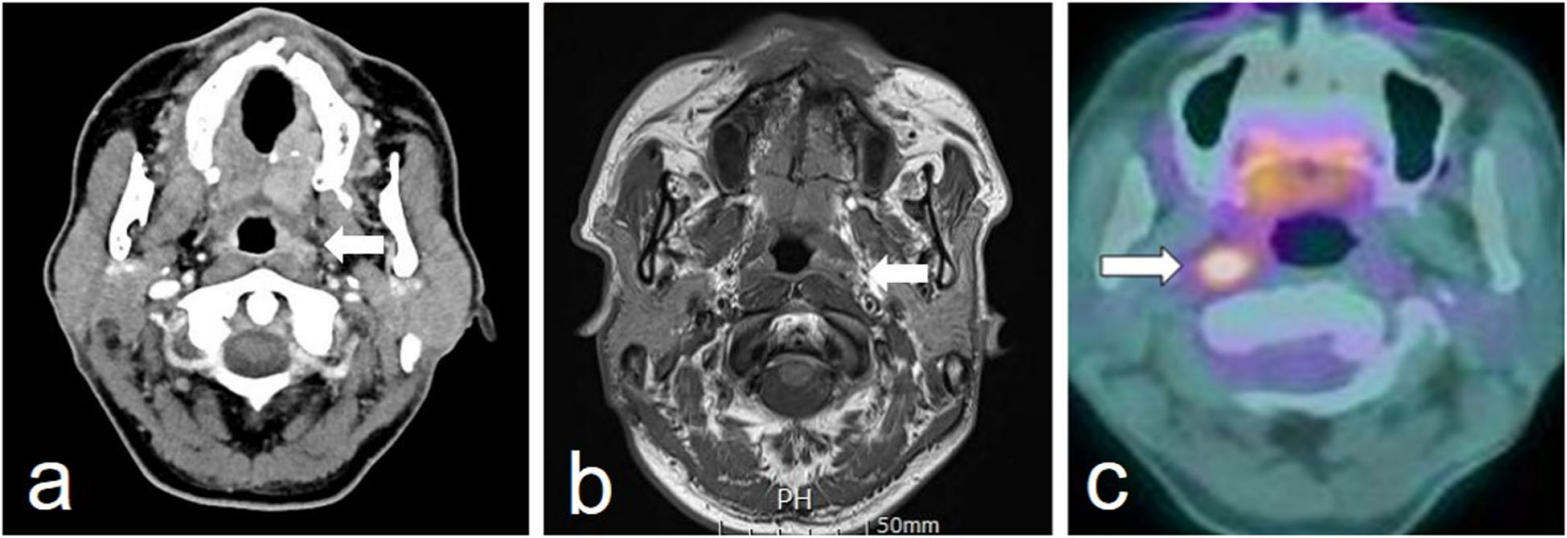
Representative false positive imaging findings of retropharyngeal lymph node. **a** Contrast-enhanced computed tomography shows a left false positive retropharyngeal lymph node (*arrow*). **b** T1-weighted gadolinium-enhanced magnetic resonance imaging shows a left false positive retropharyngeal lymph node (*arrow*). **c** Positron emission tomography-computed tomography shows an abnormal ^18^fluorine-fluorodeoxyglucose uptake with a standardized uptake value of 3.2, on a right retropharyngeal lymph node (*arrow*)

Images were interpreted to distinguish between the presence and absence of RPLN metastasis using the criteria outlined in the most recent reports [12–18]. In CT and MRI, a node in the lateral retropharyngeal region was considered to be metastatic when the minimal diameter in the axial plane was ≥6 mm, whereas any visible node in the medial retropharyngeal region was considered malignant [12–14]. Irrespective of size, nodes were considered metastatic when central necrosis or extracapsular tumor spread was noted (enhancement of the nodal capsule or poorly defined margin around the node) in contrast-enhanced CT or T1-weighted gadolinium-enhanced MRI (Fig. 1a and b) [3, 15, 16]. In PET-CT, abnormal FDG uptake in the retropharyngeal space greater than background activity which corresponded to nodular structures on CT or MRI was defined as RPLN metastasis (Fig. 1c). The cutoff maximum standardized uptake value (SUV) was 2.5, with SUV_max_ of < 2.5 defined as negative and SUV_max_ of > 2.5 defined as positive [9, 17, 18]. Figure 2 shows representative false positive cases on CT, MRI, and PET-CT.

### RPLN dissection

RPLN dissections was performed in accordance with the guidelines of our institute. Therapeutic RPLN dissections was performed in patients undergoing surgical treatment of head and neck cancer with imaging studies suggestive of RPLN metastasis. Elective RPLN dissections was performed in patients undergoing head and neck cancer surgery who had a high risk of occult RPLN metastasis; those with advanced carcinoma of the oropharynx and hypopharynx (T3 or T4), tumor extension to the posterior and lateral pharyngeal walls, or multiple-level neck node metastases [1, 17, 19].

RPLN dissection was performed either by transcervical approach or by mandibulotomy approach according to the primary tumor surgery. By transcervical approach, the retropharyngeal space was entered with a retractor placed between the carotid artery laterally and the pharyngeal constrictor muscle medially; then the most anterior layer of the prevertebral fascia was incised at a point immediately medial to the carotid bifurcation. The retropharyngeal fat pad was then identified, and dissection of this fat pad was continued in a cephalad direction carefully so as not to mistake cervical sympathetic ganglion for an enlarged retropharyngeal node. By mandibulotomy approach, the retropharyngeal space was entered directly after the removal of the primary tumor. Suspicious RPLNs in the surgical field and in preoperative images were all dissected and sent for histopathologic study. Tissues were fixed in unbuffered 10% formalin, embedded in paraffin, and serial 6-μm thick sections were stained using hematoxylin/eosin and PAS (Periodic Acid Schiff). Immunohistochemistry was not performed.

### Statistical analysis

Pathological findings of dissected RPLNs were compared to radiologic findings to determine the sensitivity, specificity, positive predictive value, negative predictive value, and overall accuracy of each imaging study. Analyses were conducted using IBM SPSS Statistics version 20 (IBM Corporation, Armonk, NY, USA).

## Results

Among the 123 patients, 19 (15%) were recurred cases with a history of previous treatment, while the other 104 were fresh cases. The recurred cases consisted of 11 cases of previously treated with surgical management only (range 13–59 months before PRLN dissection), and four cases with previous surgery and radiotherapy (range 12–108 months before PRLN dissection), and four cases with surgery and chemoradiotherapy (range 12–47 months before PRLN dissection). There were 109 male patients and 14 female patients, aged between 33 and 81 years (mean, 59.2 years). Final pathologic diagnosis of the primary tumor was squamous cell carcinoma in all cases. The sites of the primary tumors are shown in Table 1. Most tumors were located in the oropharynx and hypopharynx (54 and 39%, respectively). Pathologic TNM stages of the patients are shown in Table 2; most patients had stage IV disease (88%). No patient in this study showed distant metastasis in PET-CT at the time of surgery; for those who had not taken PET-CT, no evidence of distant metastasis was noted in any of the preoperative studies including chest x-ray or CT, gastroduodenoscopy, colonoscopy, etc. The surgical approaches performed included laryngopharyngectomy in 42 patients (34%), mandibular splitting in 39 (32%), transcervical approach in 23 (19%), and lateral pharyngotomy approach in 19 (15%).

**Table 1:** Primary cancer sites and RPLN status of the patients

Primary site	Among 123 patients No. (%)	Among 43 RPLN positive patients No. (%)
**Oral cavity**	7 (6%)	4 (9%)
**Oropharynx**	66 (54%)	27 (63%)
**Hypopharynx**	48 (39%)	12 (28%)
**Larynx**	1 (1%)	0
**Sinonasal**	1 (1%)	0

Abbreviations: *RPLN* Retropharyngeal lymph node

**Table 2:** Pathologic TNM staging of the patients

Pathologic stage		Among 123 patients No. (%)	Among 43 RPLN positive patients No. (%)
**T classification**	T0	6 (5%)	5 (12%)
	T1	8 (7%)	5 (12%)
	T2	45 (37%)	12 (28%)
	T3	20 (16%)	8 (19%)
	T4a	40 (33%)	10 (23%)
	T4b	4 (3%)	3 (7%)
**N classification**	N0	19 (15%)	1 (2%)
	N1	10 (8%)	3 (7%)
	N2a	2 (2%)	0
	N2b	56 (46%)	22 (51%)
	N2c	30 (24%)	16 (37%)
	N3	6 (5%)	1 (2%)
**TNM stage**	I	1 (1%)	0
	II	7 (6%)	0
	III	7 (6%)	0
	IVa	99 (81%)	38 (88%)
	IVb	9 (7%)	5 (12%)

Abbreviations: *TNM* Tumor-node-metastasis, *RPLN* Retropharyngeal lymph node

Therapeutic RPLN dissection was performed in 42 patients (34%) and elective RPLN dissection in the other 81 patients (66%). Pathology reports confirmed cancer metastasis to RPLNs in 43 out of 123 patients (35%). The rate of positivity in pathologic study was 83.3% among patients who underwent therapeutic RPLN dissection but only 9.9% among patients who underwent elective RPLN dissection. Primary sites in the 43 patients with confirmed metastasis to RPLN was mostly oropharynx (63%) or hypopharynx (28%) (Table 1). Among these patients, the TNM stages were IVa (88%) and IVb (12%) (Table 2). Right side RPLN metastasis was found in 23 patients (53.5%) and left side RPLN metastasis was found in 30 patients (69.8%), with ten patients (23.3%) on both sides. RPLN metastasis was medial in 5 patients (11.6%) and lateral in 40 patients (93%). The average number of RPLNs observed in pathology study in each patient was 4.2 (range, 1–8) (*n* = 123), and the average number of positive RPLNs in each positive case was 2.1 (range, 1–5 nodes) (*n* = 43).

The results of pathologic diagnosis of RPLNs according to CT, MRI, and PET-CT findings are shown in Table 3. The sensitivity of CT was the lowest and that of PET-CT the highest, while the specificities were similar (range, 93.3–93.8%) among the three imaging modalities (Table 4). Interestingly, the sensitivity values were lower than the specificity values in all three imaging modalities. The positive and negative predictabilities of the three modalities did not differ much, ranging between 83 and 89%. The overall accuracy in detecting RPLN metastasis was the highest for PET-CT and the lowest for CT. Considering all the three imaging modalities in combination (“radiologically positive” diagnosis was made when positive finding was found in any of the three modalities in a patient, *n* = 74), our study resulted in an overall sensitivity of 89.7%, specificity of 91.1%, positive predictive value of 86.7%, negative predictive value of 93.2%, and accuracy of 90.5% in detecting RPLN metastasis of head and neck squamous cell carcinoma.

**Table 3:** CT, MRI, and PET-CT findings and their concordance with pathologic findings for RPLN metastasis

Imaging modalities	Total No.	Pathologic findings	
		Positive RPLN	Negative RPLN
**CT findings**			
**Positive RPLN**	33	28	5
**Negative RPLN**	90	15	75
**MRI findings**			
**Positive RPLN**	37	32	5
**Negative RPLN**	86	11	75
**PET-CT findings**			
**Positive RPLN**	27	24	3
**Negative RPLN**	47	5	42

Abbreviations: *CT* Computed tomography, *MRI* Magnetic resonance imaging, *PET-CT* Positron emission tomography-computed tomography, *RPLN* Retropharyngeal lymph node

**Table 4:** Accuracies of CT, MRI, and PET-CT in detecting RPLN metastasis confirmed by pathologic findings

Imaging modalities	Sensitivity (%)	Specificity (%)	PPV (%)	NPV (%)	Accuracy (%)
**CT** (*n* **=** 123)	**65.1**	**93.8**	**84.8**	**83.3**	**83.7**
**MRI** (*n* **=** 123)	**74.4**	**93.8**	**86.5**	**87.2**	**87.0**
**PET-CT** (*n* **=** 74)	**82.8**	**93.3**	**88.9**	**89.4**	**89.2**
**CT + MRI + PET-CT** (*n* **=** 74)	**89.7**	**91.1**	**86.7**	**93.2**	**90.5**

Abbreviations: *CT* Computed tomography, *MRI* Magnetic resonance imaging, *PET-CT* Positron emission tomography-computed tomography, *PPV* Positive predictive value, *NPV* Negative predictive value

When we compared fresh cases (*n* = 104) with recurred cases (*n* = 19), the sensitivity (64.2% for fresh cases versus 66.9% for recurred cases), specificity (93.9% versus 92.8%), positive predictive value (85.4% versus 84.3%), negative predictive value (85.2% versus 83%), and accuracy (84.2% versus 83.1%, respectively) of CT were not significantly different between the two groups (all *P* > 0.05, student *t* test). The MRI sensitivity (74.9% for fresh cases versus 70.1% for recurred cases), specificity (93.9% versus 91.1%), positive predictive value (85.8% versus 87.9%), negative predictive value (88.2% versus 86.1%), and accuracy (87.2% versus 86.1%), and the PET-CT sensitivity (82.8% versus 82%), specificity (93.8% versus 92.9%), positive predictive value (89.1% versus 88.7%), negative predictive value (89.5% versus 89.1%), and accuracy (89.3% versus 88.8%, respectively) of the two groups neither showed significant differences (all *P* > 0.05).

## Discussion

RPLNs lie within a fat pad located behind the posterior wall of the pharynx and in front of the prevertebral fascia. RPLNs can be divided into the medial and lateral groups. The medial group lies behind the pharyngeal midline at the level between the first and third cervical vertebrae. The lateral group is better known as the “nodes of Rouviere” and lies medial to the internal carotid artery and sympathetic chain, at the level of the atlas. Afferent lymphatic vessels originate from the mucosa of the nasopharynx, tonsillar fossa, and oropharyngeal and hypopharyngeal walls, while efferent lymphatic vessels travel to the upper jugular chain and the posterior triangle of the neck [1]. The reported incidence of RPLN metastasis of primary squamous cell carcinoma of the head and neck is between 4.4 and 44.1% [20]. The highest incidence of RPLN metastasis, between 29.1 and 88.6%, is reported for nasopharyngeal carcinoma [20].

Patients with RPLN metastasis have poor prognosis [1–4]. Our institute has previously reported that oropharyngeal squamous cell carcinoma patients with RPLN metastasis have a significantly lower disease-specific survival rate than those without RPLN metastasis [19]. A positive RPLN on a preoperative image is an independent risk factor associated with RPLN metastasis in multivariate analysis [19]. However, there is still no convincing evidence that RPLN dissection improves survival or regional control in head and neck squamous cell carcinoma [6].

RPLNs are generally small, even with metastasis (< 1.5 cm) [6]. Normal medial RPLNs are too small to be visualized by either CT or MRI. Their visualization is generally considered a sign of pathology [5, 21]. In contrast, adenopathy in the lateral retropharyngeal space can be visualized, whether it is benign or metastatic. Lateral RPLNs are seen on CT in two-thirds of healthy patients, ranging in size from 3 to 7 mm [21]. The diagnosis of RPLN metastasis in imaging studies is based on the presence of necrosis, extracapsular spread, or the size of the node. Central necrosis is reportedly the most accurate CT criterion for lymph node metastasis [22]. Extranodal tumor spread diagnosed with CT has a nearly 100% sensitivity for nodal metastasis [22], while nodal shape is not a reliable criterion [3]. The same size and shape criteria are used for CT and MRI [22]; however, the size cutoff for positive identification of RPLN metastasis varies among authors. The usual size criterion for defining abnormal enlargement of lymph nodes in other parts of the neck is 10 mm; however, the reported cutoffs for RPLNs are lower considering that RPLNs are smaller than other lymph nodes in the upper neck [1, 3, 21]. Mancuso et al. have found that the maximum RPLN diameter in normal subjects measured by CT is 5–8 mm in young adults and 3–5 mm in older adults [21]. MRI studies have revealed that normal RPLNs measure less than 4 or 4.5 mm in minimal axial diameter [23, 24]. A maximal axial diameter cutoff of 8 or 10 mm [20, 22, 23], or a minimal axial diameter of 5 mm have been used as the radiologic size criteria of RPLN metastasis [15, 23, 25]. By using microscopic examination of dissected lymph nodes, Van den Brekel et al. have estimated the accuracy of different radiologic criteria used to detect cervical lymph node metastasis in head and neck squamous cell carcinoma patients, and reported that the minimal axial diameter was the most accurate criterion (in comparison with maximal axial diameter, longitudinal diameter, location, and node shape) [3]. By using a receiver operating characteristic curve, Zhang et al. found that the most accurate size criterion was the minimal axial diameter of 6 mm, which showed an accuracy of 87.5% [14]. Using MRI, Li et al. found that the minimal axial diameter of 6 mm is a better prognostic predictor of survival of nasopharyngeal cancer patients in comparison with 5 mm [13]. The minimal axial diameter of RPLN is positively correlated with the SUV of PET-CT (*r* = 0.832, *P* < 0.001) [26]. In this study, we used short-axis diameter of 6 mm as a size criterion of an abnormal RPLN on CT or MRI.

With improving technology, CT, MRI, and PET-CT now play an essential role in the management of head and neck cancer. Metastasis to RPLNs is difficult to determine clinically; however, recent techniques including sectional imaging have improved sensitivity in detecting nodal metastasis. Contrast-enhanced CT has been considered to be the best modality for identification of lymph node metastasis; however, suspicion of the presence of a metastatic RPLN based on CT alone is not enough [6]. MRI, which has advantages including the ability to identify smaller nodes and to distinguish nodes from the primary tumor, reportedly allows better detection of RPLN metastasis than CT [27, 28]. FDG-PET is more accurate than CT or MRI in detecting cervical lymph node metastasis in head and neck squamous cell carcinoma [29]. PET-CT reportedly offers higher sensitivity and specificity than MRI because PET-CT can detect increased glucose uptake [25]. The sensitivity of PET for locating nodal metastases has been reported to be 100% for nodes greater than 1 cm, 83% for nodes sized 6–10 mm, and 23% for nodes sized 5 mm or less [29]. For FDG PET, Kim et al. reported a sensitivity of 100%, specificity of 98.9%, and overall accuracy of 99% in evaluating the post-radiotherapy neck node status in head and neck cancer patients [30].

The sensitivity of CT for detecting metastatic RPLNs varies greatly among studies that used pathologic findings as a reference standard, from no better than 50% reported by Morrissey et al. [6], and up to 100% sensitivity and specificity reported by Okumura et al. [10]. Kato et al. reported the sensitivity of CT as 37–60% and that of MRI as 90–97% [31]. They revealed that MRI was superior to CT for the detection of metastatic RPLNs, but the study was based on the follow-up MRI data that lacked pathologic confirmation. PET-CT, when used in combination with CT and MRI, showed 88.9% sensitivity, 85.7% specificity, and 86.7% overall accuracy in detecting metastatic RPLNs [9].

The reported diagnostic values of the three imaging modalities vary widely. Our study is the first one to evaluate their diagnostic values together with pathologic confirmation. The sensitivity of CT was lower than that of the other imaging modalities, which indicates that CT alone is not sufficient to detect RPLN metastasis. The diagnostic accuracy and sensitivity of MRI in our study were slightly higher than those of CT, in line with the previous reports. The sensitivity of PET-CT was higher than that of MRI or CT, but the specificity of PET-CT was the lowest. Interestingly, the sensitivities of the three imaging modalities were lower than their specificities, even in PET-CT (however, MRI findings can be rather suboptimal on this point). This implies that a lower size cutoff may improve sensitivity. Metastasis in RPLNs was revealed in about 10% of the patients who underwent elective RPLN dissection, indicating that close attention should be paid to RPLNs even if preoperative imaging does not suggest positive findings, especially in patients with advanced head and neck cancer. Importantly, when the three imaging modalities were all used preoperatively, the sensitivity and diagnostic accuracy were the greatest, meaning that we can minimize missed diagnoses of RPLN metastasis by using all three modalities for preoperative imaging studies. The difference in the accuracies between new and recurrent cases was not significant, which may be due to the advancement of imaging technology or the strict inclusion criteria for recurrent cases in our study: we included only those who had prior therapy at least 1 year before the recurrence, because the accuracy of imaging studies can be affected by prior treatments.

One limitation of this study is its retrospective nature. Selection bias can be another limitation, as not all the patients with head and neck squamous cell carcinoma were explored for RPLNs (i.e., those who had no indications for RPLN dissection could have been overlooked). However, our study included even patients without radiologic evidence of RPLN metastasis who underwent elective dissection (i.e., high-risk patients); such patients are not usually included in similar studies, and their inclusion may decrease the bias. Micrometastases could have been missed on routine histopathological examination; therefore, the real sensitivity may be lower. Lastly, the diagnostic cutoff values for size and SUV used in this study can be debated, and their values could influence the results. The diagnostic accuracies of different cutoff values for size and SUV were not assessed in this study; to improve reliability, various cutoff values should be investigated. Further prospective studies with larger sample sizes that would include various cutoff values for size and SUV, or use the three imaging modalities for each node positive on histology, could provide more precise information that would be beneficial to this field of medicine.

## Conclusions

Diagnostic accuracies of CT, MRI, and PET-CT in detecting RPLN metastasis of head and neck squamous cell carcinoma were examined in this study by pathologic examination of RPLNs dissected from patients. The preoperative imaging findings for RPLN metastasis offered relatively high specificity rates, but rather low sensitivity rates, suggesting that only one preoperative imaging study is not sufficient to evaluate RPLN metastasis. As the diagnostic accuracy increased when all the three imaging modalities were used together, we recommend using all three imaging modalities preoperatively to decreased the number of missed cases of RPLN metastasis and improve prognosis of head and neck squamous cell carcinoma.

## Data Availability

The dataset supporting the conclusions of this article are summarized in Tables 1, 2, and 3.

## Abbreviations

RPLN: Retropharyngeal lymph node
CT: Computed tomography
MRI: Magnetic resonance imaging
PET-CT: Positron emission tomography-computed tomography
FDG: Fluorodesoxyglucose
SUV: Standardized uptake value
